# MicroRNA-101 regulated transcriptional modulator SUB1 plays a role in prostate cancer

**DOI:** 10.1038/onc.2016.164

**Published:** 2016-06-06

**Authors:** B V S K Chakravarthi, M T Goswami, S S Pathi, A D Robinson, M Cieślik, D S Chandrashekar, S Agarwal, J Siddiqui, S Daignault, S L Carskadon, X Jing, A M Chinnaiyan, L P Kunju, N Palanisamy, S Varambally

**Affiliations:** 1 Michigan Center for Translational Pathology, University of Michigan, Ann Arbor, MI, USA; 2 Department of Pathology, University of Michigan, Ann Arbor, MI, USA; 3 Molecular and Cellular Pathology, Department of Pathology, University of Alabama at Birmingham, Birmingham, AL, USA; 4 Center for Cancer Biostatistics, Department of Biostatistics, University of Michigan, Ann Arbor, MI, USA; 5 Department of Urology, University of Michigan, Ann Arbor, MI, USA; 6 Howard Hughes Medical Institute, University of Michigan Medical School, Ann Arbor, MI, USA; 7 Comprehensive Cancer Center, University of Michigan Medical School, Ann Arbor, MI, USA; 8 Comprehensive Cancer Center, University of Alabama at Birmingham, Birmingham, AL, USA

## Abstract

MicroRNA-101, a tumor suppressor microRNA (miR), is often downregulated in cancer and is known to target multiple oncogenes. Some of the genes that are negatively regulated by miR-101 expression include histone methyltransferase *EZH2* (enhancer of zeste homolog 2), *COX2* (cyclooxygenase-2), *POMP* (proteasome maturation protein), *CERS6*, *STMN1*, *MCL-1* and *ROCK2*, among others. In the present study, we show that miR-101 targets transcriptional coactivator SUB1 homolog (*Saccharomyces cerevisiae*)/PC4 (positive cofactor 4) and regulates its expression. SUB1 is known to have diverse role in vital cell processes such as DNA replication, repair and heterochromatinization. SUB1 is known to modulate transcription and acts as a mediator between the upstream activators and general transcription machinery. Expression profiling in several cancers revealed SUB1 overexpression, suggesting a potential role in tumorigenesis. However, detailed regulation and function of SUB1 has not been elucidated. In this study, we show elevated expression of SUB1 in aggressive prostate cancer. Knockdown of SUB1 in prostate cancer cells resulted in reduced cell proliferation, invasion and migration *in vitro*, and tumor growth and metastasis *in vivo*. Gene expression analyses coupled with chromatin immunoprecipitation revealed that SUB1 binds to the promoter regions of several oncogenes such as *PLK1* (Polo-like kinase 1), *C-MYC*, serine-threonine kinase *BUB1B* and regulates their expression. Additionally, we observed SUB1 downregulated CDKN1B expression. PLK1 knockdown or use of PLK1 inhibitor can mitigate oncogenic function of SUB1 in benign prostate cancer cells. Thus, our study suggests that miR-101 loss results in increased SUB1 expression and subsequent activation of known oncogenes driving prostate cancer progression and metastasis. This study therefore demonstrates functional role of SUB1 in prostate cancer, and identifies its regulation and potential downstream therapeutic targets of SUB1 in prostate cancer.

## Introduction

Prostate cancer is the most common malignancy and the second most common cause of cancer death among men in the United States.^1^ Multiple molecular alterations have been identified in prostate cancer initiation, growth, invasion and metastasis. Further investigations are needed to understand the mechanisms of dysregulation and role in tumorigenesis of many of the dysregulated genes that are implicated in cancer. These studies will enhance the understanding of the disease process and help in developing new therapies. High-throughput gene expression profiling studies and transcriptome analyses have revealed tumor-specific gene signatures and multiple oncogenic drivers in cancers.^2, 3, 4, 5, 6, 7, 8, 9, 10^

Loss of tumor suppressor microRNAs (miRNAs or miR) is an established mechanism in cancer progression. Our analysis suggested that SUB1 homolog (*Saccharomyces cerevisiae*) (SUB1) is a target of miR-101. Our previous studies showed that genomic loci encoding miR-101 were deleted in aggressive prostate cancer leading to reduced miR-101 expression, resulting in overexpression of histone methyltransferase enhancer of zeste homolog 2 (EZH2).^11^ Apart from regulating EZH2, it has been shown that miR-101 can target other critical genes such as *COX2* (cyclooxygenase-2), *POMP* (proteasome maturation protein), *CERS6*, *STMN1*, *MCL-1* and *ROCK2*, among others.^11, 12, 13, 14, 15, 16^

In this study, we characterized SUB1 expression specifically in aggressive prostate cancer. Yeast SUB1 is biochemically identified as a stimulator of *in vitro* basal transcription that binds to single-strand DNA in the regions of transcription initiation.^17, 18^ SUB1 is a nuclear protein and is shown to have a role in various cellular processes.^19, 20, 21, 22, 23, 24^ It is substituted for replication protein A during transcription elongation.^25^ In addition to its role as transcriptional coactivator, SUB1 has been shown to repress promoter-driven transcription as well as nonspecific transcription *in vitro*.^26, 27^ Studies have reported that SUB1 interacts with distinct domains of activators such as VP16, GAL4, AP2, HIV-TAT, P53 and SMYD3 to modulate their functions.^19, 22, 28, 29, 30, 31, 32^ Previous studies indicated that the overexpression of SUB1 in a population of normal dermal multipotent fibroblasts resulted in the tumorigenic transformation of the cells, indicating its role in tumorigenesis. SUB1 has been recently found to show an upregulated level in different cancers. Expression of SUB1 was correlated with the levels of VEGF-C, VEGF-D and VEGFR-3 during the development of lymphangiogenesis and lymphatic metastasis in lung adenocarcinoma.^33^ SUB1 is also shown to have a role in non-small-cell lung cancer^34^ and astrocytoma.^35^ A recent study demonstrated that SUB1 has a role in SMYD3-mediated transactivation of growth/invasion-stimulatory genes in cancer cells.^32^

Here, we show increased SUB1 expression in prostate cancer cell lines and tissues. Through gene knockdown studies, we demonstrate that SUB1 has an important role in prostate cancer cell proliferation and invasion both *in vitro* and *in vivo*. We investigated the role of miR-101 in regulating SUB1 expression. Furthermore, our studies reveal that SUB1 can regulate several oncogenes including therapeutic targets such as *PLK1*, *BUB1B* and *C-MYC* by directly binding to their promoters. Finally, our studies indicate that SUB1-mediated oncogenic phenotype can be reversed by blocking PLK1 activity using PLK1 inhibitor.

## Results

### MiR-101 targets SUB1 and regulates its expression

**Figure 1:**
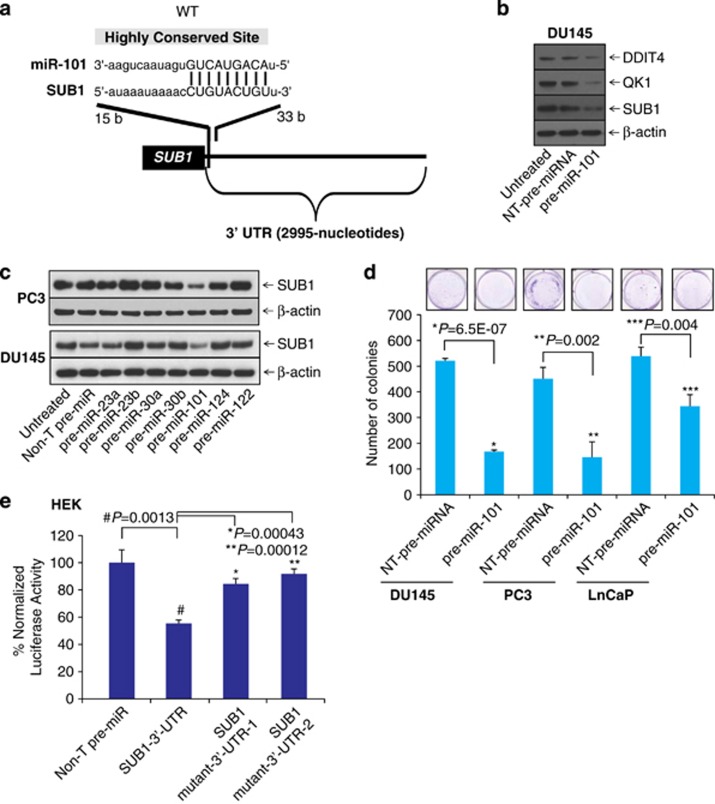
MiR-101 targets and downregulates SUB1 expression. (**a**) The predicted miR-101 binding site at the 3′-UTR of SUB1. (**b**) Immunoblot analysis showing SUB1, QK1 and DDIT4 in pre-miR-101- and control pre-miR-treated DU145 cell lysates. (**c**) Immunoblot analysis showing SUB1 protein expression in DU145 and PC3 cells treated with a panel of miRNAs. (**d**) Images of colony growth of cells either treated with non-T pre-miRNA or pre-miR-101. Quantitative data were presented in the histogram. (**e**) Luciferase reporter assay of SUB1 3′-UTR. HEK-293T cells were transfected either with pre-miR-101 or non-T pre-miR along with either SUB1 3′-UTR wild-type, mutant-1 or mutant-2 luciferase constructs. pRL-TK vector was used as an internal control.

Several studies have revealed that miRNAs are important regulators in cellular processes, such as cell proliferation, invasion and metastasis by repressing several oncogenes.^11, 12, 13, 36, 37^ To investigate the potential targets of miR-101, we used freely available web-based miR target prediction resources: TargetScan,^38^ miRanda^39^ and Diana-microT.^40^ We identified that miR-101 could potentially target SUB1. The binding site for miR-101 at 3′-UTR (untranslated region) of SUB1 is indicated (Figure 1a). MiR-101 is known to have tumor suppressor function by targeting several oncogenes including EZH2,^11^ proteasome assembly factor POMP,^13^ COX2,^12^ and others, therefore we sought to determine its role in SUB1 regulation. We treated prostate cancer cell line DU145 with precursor miRNA, miR-101 and tested some of the potential targets. We observed downregulation of QK1 and DDIT4 protein levels along with SUB1 (Figure 1b). Quantitative real-time PCR (qPCR) analyses also showed that miR-101 downregulates *SUB1*, *DDIT4* and *STC1* mRNA levels (Supplementary Figure S1). Here we show that miR-101 targets other known players in prostate cancer DDIT4, STC1 and QK1. To find the effect on SUB1 protein levels, we treated prostate cancer cells with precursor miRs, miR-101, -23a, -23b, -30a, -30b, -124 and -122 individually and measured SUB1 protein levels. As shown in Figure 1c, miR-101-treated cells showed significant reduction in SUB1 protein levels, whereas the control and other miR precursors did not alter SUB1 expression. Further, we measured the effect of miR-101 on cell growth using a colony growth assay using DU145, PC3 and LnCaP cells (Figure 1d). To determine whether miR-101 directly binds SUB1 3′-UTR and regulates it, HEK-293T cells were co-transfected with miR-101 and pMir-REPORT-SUB1 3′-UTR plasmids. MiR-101 showed substantial reduction in luciferase reporter activity compared with non-targeting (Non-T) control miR (Figure 1e). This effect is reversed by mutating miR-101 target site (Supplementary Figures S2a and b). These results indicate that SUB1 is a direct target of miR-101.

### Transcriptional coactivator SUB1 expression in prostate cancer

**Figure 2:**
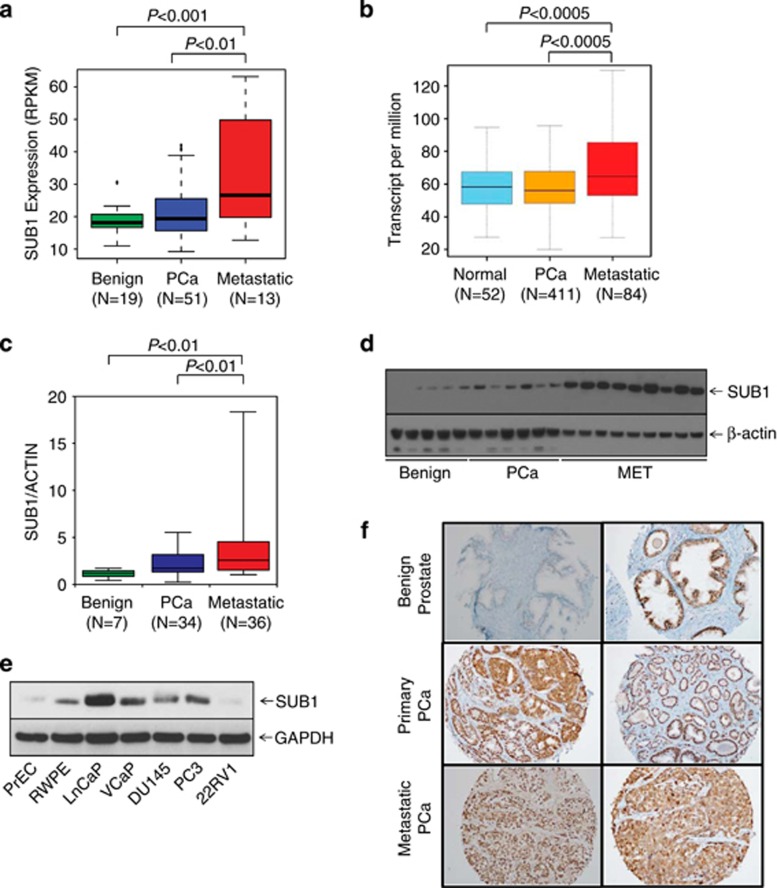
SUB1 is overexpressed in aggressive prostate cancer. (**a**) *SUB1* gene expression from next-generation RNA sequencing (RPKM (log 2)) data from benign, prostate carcinoma (PCa) and metastatic prostate cancer (MET) tissues. (**b**) Expression of *SUB1* in normal prostate, primary tumor and metastatic tumor samples from TCGA. (**c**) qPCR of *SUB1* using RNA from benign, PCa and MET tissues. (**d**) *SUB1* protein expression by immunoblot analysis of prostate tissue extracts using SUB1 antibody. β-Actin was used as a loading control. (**e**) Immunoblot analysis of SUB1 in prostate cancer cell lines. GAPDH was used as a loading control. (**f**) Immunohistochemical analysis of SUB1 in benign prostate epithelia (top), primary PCa (middle) and metastatic PCa (bottom).

The expression profiling and transcriptome sequence analysis showed upregulation of SUB1 in metastatic prostate cancer (Figure 2a). Moreover, The Cancer Genome Atlas (TCGA) data show that SUB1 is overexpressed in metastatic prostate adenocarcinoma (Figure 2b). To validate this observation, we performed qPCR using RNA from multiple prostate cancer and benign tissues and confirmed increased expression of SUB1 in metastatic prostate cancer tissues relative to benign prostate samples (Figure 2c). Immunoblot analysis using SUB1-specific antibody (Figure 2d) indicated SUB1 protein overexpression. Similarly, elevated levels of SUB1 protein was observed in metastatic prostate cancer cell lines relative to benign cell lines (Figure 2e). Additionally, we investigated SUB1 protein expression in a large number of prostate cancer samples by immunohistochemical analysis, which showed weak or no reactivity in many benign tissues but stronger staining in aggressive prostate cancer tissue and metastatic prostate tumors (Figure 2f).

### SUB1 expression is essential for prostate cancer cell proliferation and invasion

**Figure 3:**
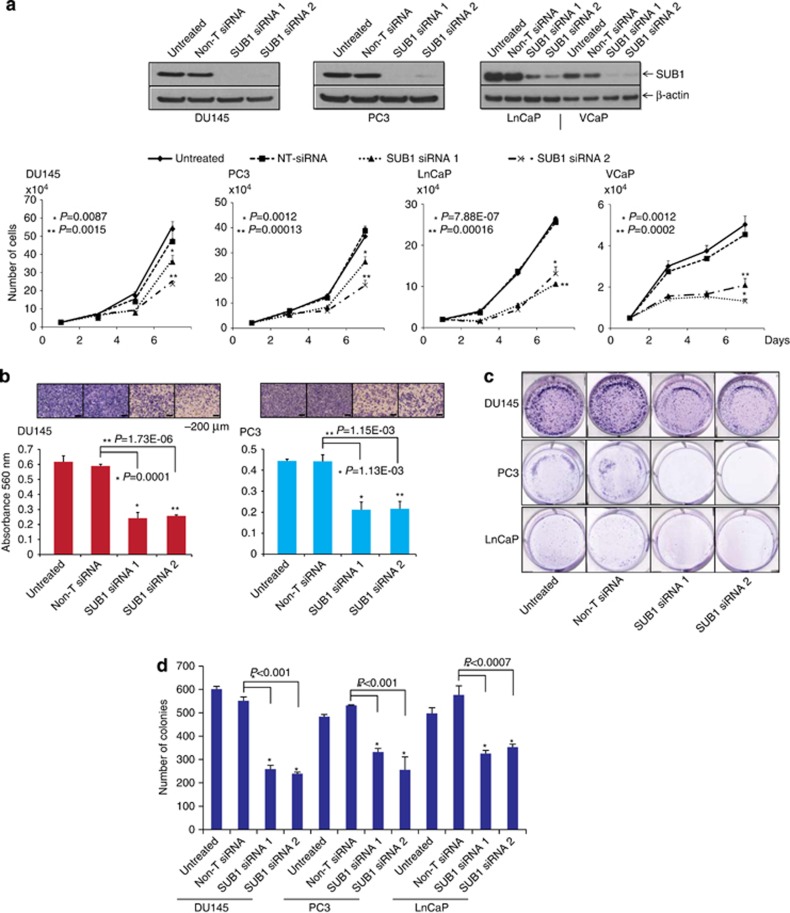
SUB1 is essential for prostate cancer cell proliferation and invasion. (**a**) Transient knockdown of SUB1 in prostate cancer cell lines reduces prostate cancer cell proliferation. Immunoblot analysis of protein using lysates from prostate cancer cell lines DU145, PC3, LnCaP and VCaP treated with two specific and independent SUB1 siRNA duplexes. β-Actin was used as a loading control. Cell proliferation assay of these cells transfected with either SUB1 siRNA duplex or Non-T siRNA control. (**b**) Knockdown of SUB1 reduces DU145 and PC3 cell invasion. Boyden chamber Matrigel invasion assay was performed using DU145 or PC3 cells in which SUB1 was transiently knocked down using two independent SUB1 siRNA duplexes. Non-T siRNA-treated cells served as control. Invaded cells were stained with crystal violet and the absorbance was measured at 560 nm. (**c**) Representative images of colony formation assay. (**d**) Quantification of colonies formed. The colony formation efficiency was significantly reduced in both SUB1 siRNA1- and 2-treated cells as compared with untreated and Non-T siRNA controls.

To determine the functional significance of SUB1 expression in prostate cancer, we perturbed SUB1 levels in prostate cells and investigated the effect of this modulation on cell proliferation, migration and invasion. We used both transient RNA interference and stable knockdown strategies targeting SUB1 in aggressive prostate cancer cell lines DU145 and PC3 and hormone-responsive LnCaP and VCaP cells. The efficiency of SUB1 knockdowns were confirmed by immunoblot (Figure 3a) and qPCR (Supplementary Figures S3a–g) analyses. We observed significant decrease in cell proliferation upon transient knockdown of SUB1 compared with control cells transfected with non-T small interfering RNAs (siRNAs) (Figure 3a). Next, we tested cell motility after stable SUB1 knockdown in prostate cancer cells using wound healing assay. The efficiency of SUB1 stable knockdowns were confirmed by immunoblot (Supplementary Figure S4a). SUB1 knockdown showed a wider wound area 24 h post wound generation relative to control cells, the delayed time to heal indicating an inability of SUB1 knockdown cells to migrate (Supplementary Figures S4b and c). Additionally, SUB1 knockdown in DU145 and PC3 reduced the invasive potential of these cells as assessed by Boyden chamber Matrigel invasion assay (Figure 3b). Further we tested for colony formation after transient knockdown of SUB1 and colonies were quantified (Figures 3c and d). Taken together, these observations demonstrate the involvement of SUB1 in the proliferation, migration, invasion and colony formation of prostate cancer cells *in vitro*.

### SUB1 modulates gene expression in prostate cancer

**Figure 4:**
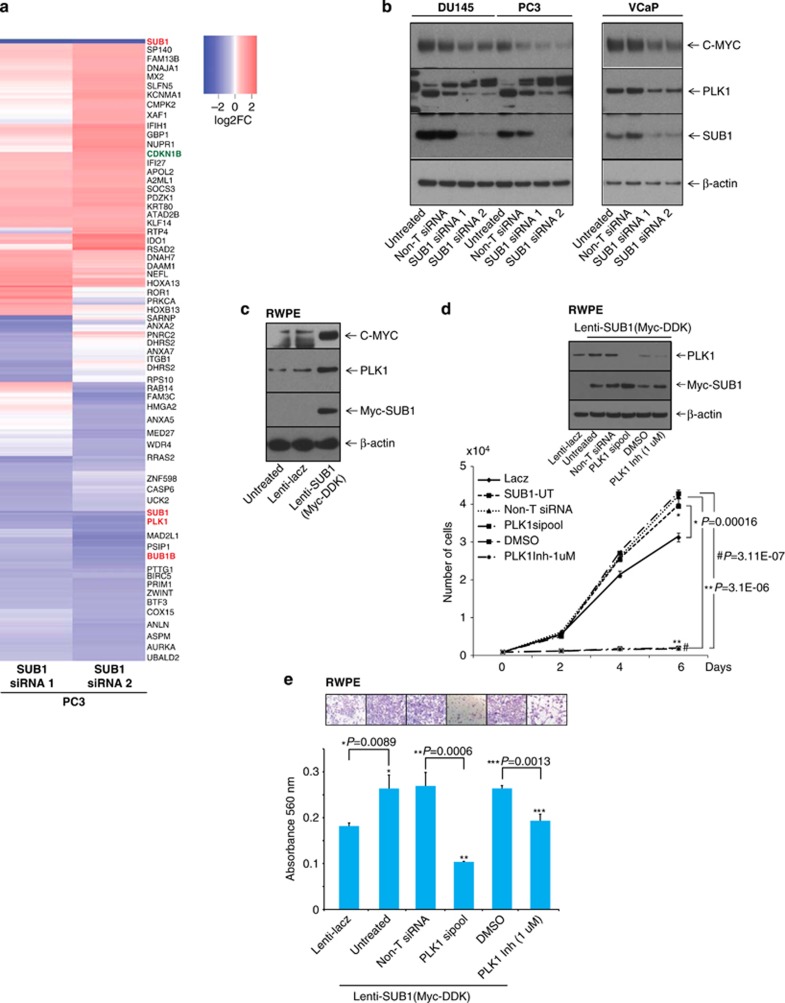
SUB1 modulates PLK1 expression in prostate cancer cells. (**a**) Heatmap of select genes in stable SUB1 knockdown PC3 cells. (**b**) Immunoblot analysis showing SUB1, PLK1 and C-MYC in prostate cancer cells after transient knockdown of SUB1. (**c**) Immunoblot analysis showing Myc-DDK-tagged SUB1, PLK1 and C-MYC in stable RWPE-SUB1- (Myc-DDK-tagged) overexpressing cells. (**d** and **e**) Immunoblot, cell proliferation and Matrigel invasion assays were performed using lenti-lacZ or lenti-SUB1 cells. Untreated cells, non-T siRNA, PLK1-specific siRNA SMARTpool-treated cells or in the presence of PLK1 inhibitor (volasertib (BI6727)) were used. Invaded cells were stained with crystal violet and the absorbance was measured at 560 nm. Inset, photomicrographs of invaded cells.

To evaluate SUB1-mediated effects in prostate cancer progression, we performed gene expression analysis using RNA from SUB1 knockdown prostate cell lines. We identified multiple genes that were modulated upon SUB1 knockdown including *PLK1*, *C-MYC*, *BUB1B* and *CDKN1B*, among others (Figure 4a). PLK1 and C-MYC are known to have a role in cell proliferation, invasion and metastasis.^41, 42^ We validated the activation of CDKN1B and the downregulation of PLK1, C-MYC and BUB1B, both at the mRNA and protein levels upon SUB1 knockdowns (Supplementary Figures S5a–h, Figure 4b and Supplementary Figures S6a–c) and observed induction of these genes upon SUB1 overexpression in RWPE cells (Figure 4c). TCGA data show that *PLK1* and *BUB1B* are overexpressed in metastatic prostate adenocarcinoma (Supplementary Figures S7a and b). Additionally, we validated both *PLK1* mRNA and protein levels across benign, prostrate carcinoma and metastatic prostate cancer tissues by qPCR and immunoblot analysis, respectively (Supplementary Figures S7c and d), which confirmed the direct correlation between SUB1 and PLK1. Additionally, *SUB1* and *PLK1* mRNA levels are correlated in prostate cancer cell lines (Supplementary Figures S7e and f). Further, we generated stable SUB1-expressing RWPE cells using lentivirus (Supplementary Figure S8). Overexpression of SUB1 resulted in increased cell proliferation (Supplementary Figure S8 and Figure 4d) and invasion (Figure 4e). Furthermore, SUB1 overexpression elevated PLK1 and C-MYC expression, and reduced CDKN1B expression (Figure 4c and Supplementary Figure S6c), showing SUB1 dysregulation triggers alterations in critical oncogenes and tumor suppressors in prostate cancer. To verify the role of PLK1 in cell proliferation and invasion, we treated stable SUB1-overexpressing RWPE cells with PLK1 siRNA SMARTpool or PLK1 inhibitor (volasertib (BI6727)) and analyzed Myc-DDK-tagged SUB1 and PLK1 (Figure 4d inset). Both PLK1 knockdown and PLK1 inhibitor decreased cell proliferation (Figure 4d) and Matrigel invasion (Figure 4e). Thus, these consolidated observations underscore a downstream role for PLK1, C-MYC and CDKN1B in SUB1-mediated prostate cancer cell proliferation and invasion.

**Figure 5:**
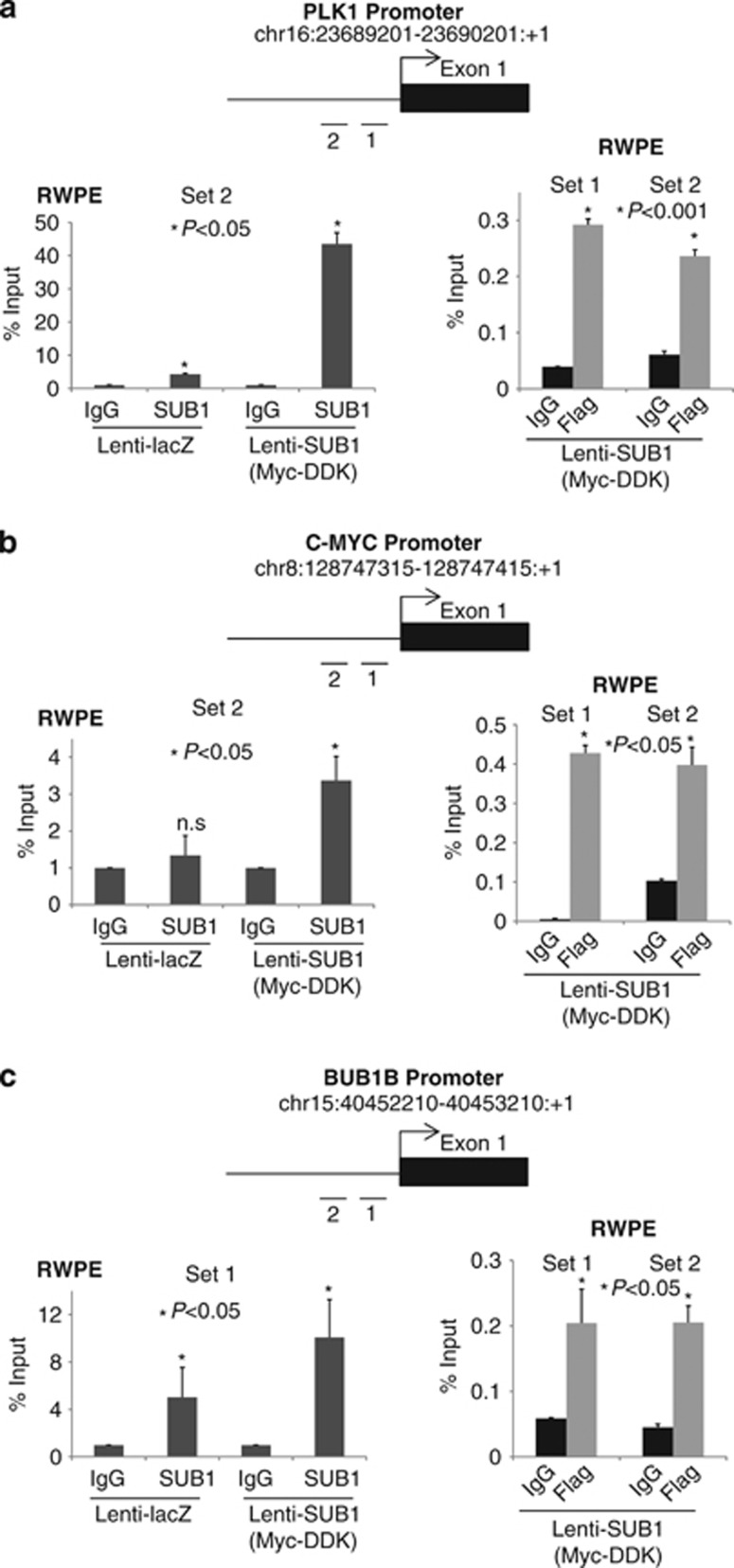
SUB1 transactivates gene expression by directly binding to specific motifs of their promoter regions. ChIP-PCR analysis for the SUB1 occupancy on (**a**) *PLK1*, (**b**) *C-MYC* and (**c**) *BUB1B* promoters in SUB1- or lacZ-overexpressing RWPE cells. ChIP was performed using antibodies against SUB1-, Myc-DDK-tagged SUB1 and a control IgG. Inset: Schematic representation of the respective genomic regions showing gene and amplicon positions.

Earlier studies suggest the importance of SUB1 in regulating transcription *in vivo*. For example, SUB1 enhances transcriptional activation by the activators GCN5 and HAP4 in yeast,^43^ and stimulates transcription *in vitro* with diverse kinds of activators, including SMYD3,^32^ VP16,^24, 44^ BRCA-1^45^ and octomer transcription factor-1,^46^ possibly by facilitating assembly of the preinitiation complex. First, we compared motif occurrences within 500 bp upstream regions of downregulated genes and undifferentially expressed genes (when SUB1 is knocked down) using a computer program called CLOVER.^47^ Next, to validate the binding of SUB1 to PLK1, BUB1B and C-MYC promoters, we conducted chromatin immunoprecipitation (ChIP) assays using commercially available anti-SUB1 or -DDK antibodies in stable RWPE cells overexpressing lacz or Myc-DDK-tagged SUB1. As expected, SUB1 is enriched at PLK1, C-MYC and *BUB1B* promoter regions (Figures 5a–c). These data demonstrate that PLK1, C-MYC and BUB1B promoters are transcriptionally activated by SUB1 in prostate cancer.

### SUB1 has a role in prostate tumor growth and metastasis

**Figure 6:**
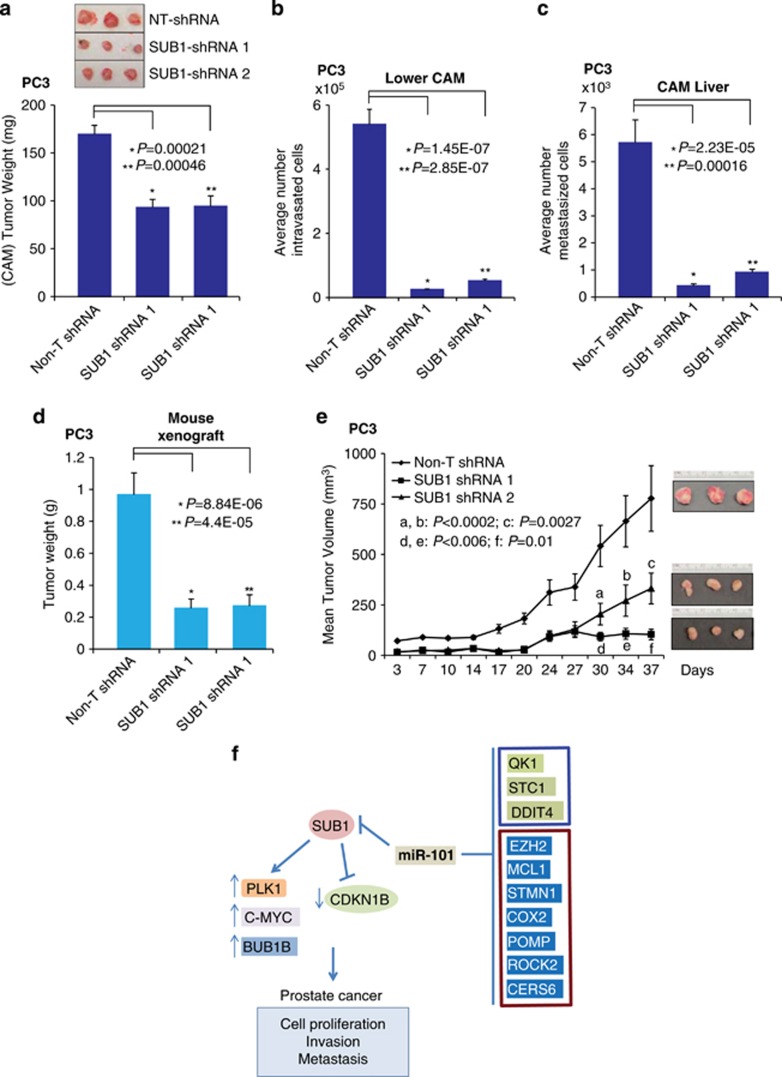
SUB1 is required for prostate tumor growth *in vivo*. (**a**) Tumor growth of stable SUB1 knockdown PC3 prostate cancer cells or control non-T shRNA PC3 cells in the chick CAM tumor assay. Extraembryonic tumors were harvested and weighed after 72 h of postimplantation of cells. Inset: Photomicrographs of CAM tumors (**b**) and (**c**) SUB1 knockdown reduces metastasis of PC3 cells in the CAM models. Cells that metastasized to the lower CAM and liver of chicken embryos were quantified using human Alu-specific PCR. (**d**) SUB1 knockdown in PC3 cells inhibits tumor growth in a mouse xenograft model. Plot of mean tumor volume at indicated time points for mice inoculated with non-T shRNA (solid line with filled diamonds) or two independent SUB1 stable knockdown shRNA 1 (solid line with filled squares) and 2 (solid line with filled triangles) cells. Inset: photomicrographs of xenograft tumors (**e**) Tumor weights of corresponding mouse xenograft models. *n=*8 mice per group. (**f**) Proposed model of SUB1 and miR-101 regulation in prostate cancer progression. SUB1 has a role in cell proliferation, invasion, metastasis and tumor growth, and is regulated by miR-101. Additionally, our study showed that miR-101 downregulates STC1, DDIT4 and QK1 expression (blue box). Additional known targets of miR-101 from published literature are also shown (brown box).^11, 12, 13, 14, 15, 16^

To demonstrate the role of SUB1 on tumor growth *in vivo*, we used a chick chorioallantoic membrane (CAM) assay and measured spontaneous metastasis, including local invasion, intravasation and metastasis to distant organs. CAM assay was performed as described previously,^37, 48^ using prostate cancer PC3-SUB1 knockdown cells. Depletion of SUB1 resulted in significantly reduced tumor weight compared with non-target short hairpin RNA (shRNA)-transfected control cells (Figure 6a). SUB1 knockdown in PC3 cells impaired their ability to invade the CAM basement membrane and resulted in a significantly decreased number of intravasated cells in the lower CAM compared with control cells (Figure 6b). Furthermore, there was attenuation of tumor metastasis in the SUB1 knockdown group compared with the control group (Figure 6c). Next, we examined SUB1-mediated tumorigenesis in a murine PC3 xenograft model using Non-T shRNA or two independent SUB1 stable knockdown PC3 cells. Both SUB1-shRNA 1 and 2 cells showed significantly reduced tumor growth and tumor weight in mice (Figures 6d and e) relative to control animals, demonstrating that SUB1 inhibition attenuates tumor growth (CAM assay and murine xenografts) and metastasis (CAM assay) *in vivo*. These observations show that SUB1 has a role in prostate tumor growth *in vivo*.

## Discussion

In this study, we show that miR-101 regulates SUB1 expression and SUB1 has a role in prostate cancer growth. We and others have earlier shown that reduced expression of miR-101 leads to overexpression of oncogenic histone methyltransferase EZH2 in multiple tumors.^11, 13, 36, 49^ A genomic loss of miR-101 or epigenetic silencing leads to reduction in miR-101 expression in multiple cancers.^50, 51^ These observations suggest that attenuation of miR-101 expression is an important event in oncogenesis. Our investigations also demonstrate the role of SUB1 in prostate cancer cell proliferation and invasion.

SUB1 protein has important roles in various cellular processes including transcription, replication, chromatin organization, cell cycle progression, DNA damage repair and apoptosis.^20, 21, 34, 52^ Earlier, it was shown that SUB1 is amplified and overexpressed in non-small-cell lung cancer cell lines,^53^ invasive intraductal papillary mucinous neoplasm of the pancreas^54^ and in carcinomatoses.^55^ Additionally, it has been shown that exogenous overexpression of SUB1 could induce the transformation of a population of normal dermal multipotent fibroblasts to acquire malignant characteristics of anchorage-independent growth *in vitro* and tumorigenicity in nude mice.^56^ Additionally, it was reported that SUB1 is upregulated in esophageal squamous cell carcinoma and its absence increased radiosensitivity of esophageal squamous cell carcinoma cells, and suppressed non-homologous end-joining activity via downregulation of XLF.^52^ Targeting coactivators and transcription factors through chemical inhibitors has been challenging.^57, 58, 59, 60^

Although earlier studies showed the role of SUB1 in cancer, its mechanistic insight in oncogenesis is not fully understood. The present study shows an increased expression of SUB1 in prostate cancer. Moreover, it is involved in tumorigenesis through the activation of PLK1, BUB1B and C-MYC, and repression of CDKN1B during cancer progression. These proteins are involved in several cellular processes including cell proliferation, cell cycle and tumorigenesis.^41, 42, 61, 62^ The oncoprotein PLK1 is known to have a role in critical cell cycle events and acts in concert with cyclin-dependent kinase 1-cyclin B1 and Aurora kinases.^63^ Moreover, in cancer these kinases are often dysregulated, promoting uncontrolled cell proliferation and aberrant cell division.^63, 64^ The PLK family members have been associated with poor prognosis, which lead to enhanced interest as promising targets for anticancer drug development.^65^ In our study, we demonstrated that SUB1 positively regulated *PLK1* expression at the transcriptional level. Furthermore, we investigated the potential role of SUB1-induced PLK1 in prostate cancer invasion by using PLK1-specific siRNA pool or inhibitor volasertib. The PLK1 inhibitor volasertib attenuated stable RWPE-SUB1 cells ability to proliferate as well as to invade through Matrigel *in vitro*. Moreover, it is currently the most clinically advanced inhibitor.^66^ Studies in various cancer cell lines (prostate, lung, colon, melanoma, hematopoietic malignancies and urothelial tumors) demonstrated that volasertib inhibits cell division leading to cell death.^67, 68, 69, 70^ Additionally, we also observed that SUB1 also regulates BUB1B.

In summary, here we show that reduced miR-101 expression results in transcriptional coactivator SUB1 overexpression (Figure 6f). SUB1 triggers increased cell proliferation, invasion, metastasis and modulates expression of several including PLK1, BUB1B, C-MYC and CDKN1B. Finally, SUB1-mediated oncogenic event can be alleviated using PLK1 siRNA or inhibitor. This study shows SUB1 overexpression in aggressive prostate cancer and reveals therapeutic options to block SUB1-mediated oncogenesis.

## Materials and methods

### Cell lines

Prostate cancer cell lines DU145, PC3 and LnCaP were grown in RPMI-1640 (Life Technologies, Carlsbad, CA, USA), whereas VCaP was grown in Dulbecco's modified Eagle's medium with penicillin–streptomycin (100 U/ml) and 10% fetal bovine serum (Invitrogen, Carlsbad, CA, USA) in 5% CO_2_ cell culture incubator. The HEK293 (ATCC), RWPE-1 (henceforth referred as RWPE; ATCC, Manassas, VA, USA) cells were grown in their respective medium as specified by the suppliers. Lentiviruses were generated by the University of Michigan Vector Core (Ann Arbor, MI, USA). Prostate cancer cells were infected with lentiviruses expressing SUB1 shRNA or Non-T shRNA controls and stable cell lines were generated by selection with 1 μg/ml puromycin (Life Technologies).

### Benign and tumor tissues

In this study, we used tissues from clinically localized prostate cancer patients who underwent radical prostatectomy. Samples were also obtained from androgen-independent metastatic prostate cancer patients from a rapid autopsy program through the University of Michigan Prostate SPORE Tissue Core as described previously.^71, 72^ The detailed clinical and pathological data are maintained in a secure relational database. The Institutional Review Board at the University of Michigan Medical School approved this study. Both radical prostatectomy series and the rapid autopsy program are part of the University of Michigan Prostate SPORE Tissue Core.

### Gene expression from TCGA

The patients clinical data for prostate adenocarcinoma were downloaded using TCGA assembler.^73^ However, downloaded data comprised of only tumor pathologic and node pathologic information. Thus, based on tumor pathologic and node pathologic data as per 'https://cancerstaging.org/references-tools/quickreferences/Documents/ProstateSmall.pdf', samples were categorized into primary and metastatic tumor. Afterwards, level3 TCGA RNA-seq data (including raw_read_count and scaled_estimate for each sample) for all primary tumor, metastatic tumor and matched normal samples were downloaded using TCGA assembler. Transcript per million values for each gene was obtained by multiplying scaled_estimate by 1 000 000. Boxplot was generated using R (https://cran.r-project.org/).

### Immunohistochemistry

Benign and prostate cancer tissues were obtained from the radical prostatectomy series at the University of Michigan and from the Rapid Autopsy Program, both part of the University of Michigan Prostate SPORE programs, through appropriate informed consent. Institutional Review Board approval was obtained to procure and analyze the tissues used in this study. Immunohistochemistry was carried out to evaluate SUB1 expression using rabbit polyclonal antibody against SUB1 (Novus Biologicals, Littleton, CO, USA; cat. no. NBP1-82454). Immunohistochemistry was performed using an automated protocol developed for the DISCOVERY XT automated slide staining system (Ventana Medical Systems Inc., Tucson, AZ, USA) using Ultramap anti-rabbit HRP (cat. no. 760-4315; Ventana Medical Systems Inc.) and was detected using ChromoMap DAB (cat. no. 760-159; Ventana Medical Systems Inc.). Hematoxylin II (cat. no. 790-2208; Ventana-Roche, Tucson, AZ, USA) was used as the counterstain. The study pathologist Dr Kunju (PK) evaluated the immunohistochemical staining.

### Immunoblot analyses

Antibodies used in the study are listed in Supplementary Table S1. All antibodies were used at dilutions optimized in our laboratory. For immunoblot analysis, 10 μg protein samples were separated on a sodium dodecyl sulfate–polyacrylamide gel electrophoresis and transferred onto Immobilon-P PVDF membrane (EMD Millipore, Billerica, MA, USA). The membrane was incubated for 1 h in blocking buffer (Tris-buffered saline, 0.1% Tween (TBS-T), 5% nonfat dry milk), followed by incubation overnight at 4 °C with the primary antibody. After a wash with TBS-T, the blot was incubated with horseradish peroxidase-conjugated secondary antibody and signals were visualized by Luminata Crescendo chemiluminescence western blotting substrate as per the manufacturer's protocol (EMD Millipore).

### Gene expression analysis

Global gene expression data was generated using RNA isolated from SUB1 siRNA knockdown PC3 and non-target control cells in profiling analysis as well as in transcriptome sequencing analysis.^6^ Expression profiling was performed using the Agilent Whole Human Genome Oligo Microarray (Agilent, Santa Clara, CA, USA) and the analysis was performed according to the manufacturer's protocol. A bioconductor package ‘agilp'^74^ was used to extract and normalize raw data from two-channel experiment arrays. Loess normalization was applied on each array. The gene expression profiling data has been deposited at gene expression omnibus (GSE74895). The differential expression of each gene was estimated by subtracting loess normalized log-2-transformed signal intensity of control sample from that of knockdown sample. Genes with absolute fold change of 2 were selected as differentially expressed genes. The heatmap.2 function of R package ‘gplots' was used to create the heat map. To measure mRNA expression levels, total RNA was isolated from prostate cancer cell lines and prostate cancer tissue samples using the RNeasy Mini Kit (Qiagen, Valencia, CA, USA). qPCR was performed as described.^37^ All primers were synthesized by Integrated DNA Technologies (Coralville, IA, USA) and PCR reactions were performed in triplicate. Primer sequences are listed in Supplementary Table S2.

### RNA interference and miRNA transfection

The siRNA duplexes (duplex 1, cat. no. SI04174835 and duplex 2, cat. no. SI00678580) used to inhibit SUB1 expression were purchased from Qiagen, and PLK1 SiGenome SMARTpool (cat. no. M-003290-01-0005) was purchased from GE Dharmacon (Lafayette, CO, USA). Precursors of respective microRNAs and negative controls were purchased from Ambion (Life Technologies). Transfections were performed either with oligofectamine or Lipofectamine RNAiMAX (Life Technologies). SUB1 shRNA (pGipz SUB1-shRNA 1 (V2LHS_85556) and SUB1-shRNA2 (V3LHS_331788)) were purchased from GE Dharmacon. Lentiviruses for these stable knockdowns were generated by the University of Michigan Vector Core. For miRNA transfection or RNA inference, we plated prostate cancer cells at 1 × 10^5^ cells per well in a 6-well plate, and after 12 h, the cells were transfected either with siRNA duplexes or miRNAs. A second identical transfection was performed 24 h later. Seventy-two hours after the first transfection, cells were harvested for RNA isolation or immunoblot analysis.

### *In vitro* overexpression

SUB1 cDNA (Origene Technologies, Rockville, MD, USA; cat. no. RC204999; Myc-DDK tagged) was cloned into Gateway expression system (Life Technologies). To generate lentiviral constructs, PCR8-SUB1 (Myc-DDK tagged) was recombined with pLenti6/V5-Dest (Life Technologies) using LR Clonase II (Life Technologies). Lentiviruses were generated by the University of Michigan Vector Core. Benign immortalized prostate cells (RWPE) were infected with lentiviruses expressing SUB1 or lacZ, and stable clones were selected with 3.5 μg/ml blasticidin (Santa Cruz Biotechnology Inc., Dallas, TX, USA).

### Cell proliferation assays

Cell proliferation was measured by cell counting. For this, transient SUB1 and PLK1 knockdown and stable cells overexpressing SUB1 were used. After 72 h of transfection using specific siRNA, the cells were trypsinized and seeded at a density of 10 000 cells per well in 24-well plates (*n*=3). Non-T siRNA-treated cells were served as controls. The stable RWPE lacZ- or SUB1-overexpressing cells were plated at the same density as mentioned earlier, trypsinized and counted at specified time points by Z2 Coulter particle counter (Beckman Coulter, Brea, CA, USA). Each experiment has been performed with three replicates per sample.

### Wound healing assay

DU145, PC3 scramble shRNA or SUB1 stable knockdown cells were seeded in 6-well plates in growth medium containing 10% fetal bovine serum and puromycin (10 μg/ml) for DU145 and PC3, and then allowed to grow to confluent monolayer. For DU145 and PC3, the cells were serum starved for 12 h and replenished with 10% fetal bovine serum-RPMI medium. The wound-induced migration was triggered by scraping the cells with a 200 ul pipette tip, washed with Dulbecco's phosphate-buffered saline and replenished with respective medium. The wound was imaged immediately (0 h) and at 24 h with an inverted phase-contrast microscope under × 4 objective.

### Matrigel invasion assay

Matrigel invasion assays were performed as described earlier.^37, 75^ Various test cells were seeded onto BD BioCoat Matrigel matrix (Corning Life Sciences, Tewksbury, MA, USA) in the upper chamber of a 24-well culture plate. The lower chamber containing respective medium was supplemented with 10% serum as a chemoattractant. After 48 h, the noninvading cells and Matrigel matrix were gently removed with a cotton swab. Invasive cells located on the lower side of the chamber were stained with 0.2% crystal violet in methanol, air-dried and photographed using an inverted microscope (× 4). Invasion was quantified by colorimetric assay. For colorimetric assays, the inserts were treated with 150 μl of 10% acetic acid and the absorbance measured at 560 nm.

### Colony formation assay

After 72 h of transfection, untreated, Non-T siRNA/miRNA- or miR-101-treated cells were counted and seeded 800 cells per one well of 6-well plates (triplicate) and incubated at 37 °C with 5% CO_2_ for 7–10 days. Colonies were fixed with 10% (v/v) ethanol for 30 min and stained with crystal violet (Sigma-Aldrich, St Louis, MO, USA) for 20 min. Then, the photographs of the colonies were taken using Amersham Imager 600RGB (GE Healthcare Life Sciences, Pittsburgh, PA, USA). Colony quantification was carried out using ImageQuant TL Colony v.8.1 software (GE Healthcare Life Sciences).

### miR reporter luciferase assays

Wild-type or mutant 3′-UTR of SUB1 were cloned into the pMIR-REPORT miRNA Expression Reporter Vector (Life Technologies). HEK293 cells were co-transfected with pre-miR-101 or controls and wild-type or mutant 3′-UTR-luc, as well as pRL-TK vector as an internal control for luciferase activity. Forty eight hours post-transfection, the cells were lysed and luciferase assays were conducted using the dual luciferase assay system (Promega, Madison, WI, USA). Each experiment was performed in triplicate.

### ChIP assays

ChIP assays were carried out with respective antibodies (Supplementary Table S1) using the EZ-Magna ChIP Kit (EMD Millipore, Billerica, MA, USA) as described.^37^ The primer sequences for the promoters analyzed are provided in Supplementary Table S3.

### Chicken CAM assay

The CAM assay for local cell invasion, intravasation, metastasis and tumor (or xenograft) formation was performed as described previously.^37, 48, 75^ After 3 days of implanting the cells in each egg, lower CAM was harvested and extraembryonic tumors were isolated and weighed. For metastasis assay, the embryonic livers were harvested on day 18 of embryonic growth and analyzed for the presence of tumor cells by quantitative human Alu-specific PCR. Genomic DNA from lower CAM and livers were prepared using Puregene DNA purification system (Qiagen) and quantification of human Alu was performed as described.^37, 48, 75^ An average of eight eggs per group was used in each experiment.

### Tumor xenograft model

All procedures involving mice were approved by the University Committee on Use and Care of Animals (UCUCA) at the University of Michigan and conform to all regulatory standards. To evaluate the role of SUB1 in tumor formation *in vivo*, we propagated stable SUB1 knockdown PC3 cells using two independent shRNAs and Non-T shRNA control cells, and inoculated 1 × 10^6^ cells subcutaneously into the dorsal flank of 5-week-old male athymic nude mice (*n*=8 for each group; Harlan Laboratories, Evigo Indianapolis, IN, USA). The tumor data obtained using scramble cells is the same as that used in an earlier study as SUB1 tumor xenograft study was conducted simultaneously using common control animals.^37^ Tumor size was measured biweekly, and tumor volumes were calculated using the formula (π/6) (*L* × *W*2), where *L* is the length and *W* is the width of the tumor. After 5 weeks, mice from different groups were killed, and then the tumors were photographed, weighed and plotted.

### Statistical analysis

To determine significant differences between two groups, Student's two-tailed *t-*test was used for all experiments except for microarray. *P*-values <0.05 were considered significant.
