# Supplementary material for: MicroRNA-101 regulated transcriptional modulator SUB1 plays a role in prostate cancer
Source: Oncogene. 2016 Jun 6;35(49):6330–40. doi: 10.1038/onc.2016.164 (PMC5140777; doi:10.1038/onc.2016.164)
Supplement: Supplementary Material [file onc2016164x1.docx]

### SUPPLEMENTARY MATERIAL

**MicroRNA-101 regulated transcriptional modulator SUB1 plays a role in prostate cancer**

Balabhadrapatruni V. S. K. Chakravarthi^1,2,3^, Moloy T. Goswami^1,2^, Satya S. Pathi^1,2,9^, Alyncia D. Robinson^3^, Marcin Cieślik^1,2^, Darshan S. Chandrashekar^3^, Sumit Agarwal^3^, Javed Siddiqui^1^, Stephanie Daignault^4^, Shannon L. Carskadon^1,2,10^_,_ Xiaojun Jing^1,2^, Arul M. Chinnaiyan^1,2,5,6,7^, Lakshmi P. Kunju^1,2^, Nallasivam Palanisamy^1,2,7,10^, Sooryanarayana Varambally^1,2,3,7,8^

^1^ Michigan Center for Translational Pathology, University of Michigan, Ann Arbor, MI, USA

^2^ Department of Pathology, University of Michigan, Ann Arbor, MI, USA

^3^ Molecular and Cellular Pathology, Department of Pathology, University of Alabama at Birmingham, Birmingham, AL, USA

^4^ Center for Cancer Biostatistics, Department of Biostatistics, University of Michigan, Ann

Arbor, MI, USA

^5^ Department of Urology, University of Michigan, Ann Arbor, MI, USA

^6^ Howard Hughes Medical Institute, University of Michigan Medical School, Ann Arbor, MI,

USA

^7^Comprehensive Cancer Center, University of Michigan Medical School, Ann Arbor, MI,

USA

^8^Comprehensive Cancer Center, University of Alabama at Birmingham, AL, USA

^9^Current Address: Huntsman Cancer Institute, University of Utah, Salt Lake City, UT,

USA.

^10^Current Address: Department of Urology, Vattikuti Urology Institute, Henry Ford Health System, Detroit, MI, USA.

**Corresponding Author**

Sooryanarayana Varambally, Ph.D., Molecular and Cellular Pathology, Department of Pathology, Comprehensive Cancer Center, University of Alabama at Birmingham, AL 35233, USA

Phone: (205) 996-1654

Email: [soorya@uab.edu](mailto:soorya@uab.edu)

**TABLES, Related to Methods**

**Table S1.** List of antibodies used in this study, Related to the Materials and methods.

| **Antibody** | **Application** | **Dilution** | **Supplier** | **Cat. No.** |
| --- | --- | --- | --- | --- |
| **SUB1** | **IB** | IB, 1:1000  IB, 1:500 | PTG Labs [Chicago, IL]  Santa Cruz Biotech [Santa Cruz, CA] | 11956-1-AP  sc-48778 |
| **SUB1** | **IB (Tissue)**  **IHC,TMA** | IB, 1:1000  TMA and IHC, 1:100 | Novus Biologicals [Littleton CO] | NBP1-82454 |
| **SUB1** | **ChIP** | ChIP, 1:100 | Novus Biologicals [Littleton CO] | NB100-59774 |
| **PLK1** | **IB** | IB, 1: 1000 | PTG Labs [Chicago, IL] | 10305-1-AP |
| **CDKN1B** | **IB** | IB, 1:1000 | BD Biosciences [San Jose, CA] | 610241 |
| **C-MYC** | **IB** | IB, 1:1000 | Cell Signaling Technology [Danvers, MA] | 13987 |
| **QK1** | **IB** | IB, 1:1000 | PTG Labs [Chicago, IL] | 13169-1-AP |
| **DDIT4/REDD1** | **IB** | IB, 1:1000 | PTG Labs [Chicago, IL] | 10638-1-AP |
| **Myc-tag** | **IB** | IB, 1:1000 | PTG Labs [Chicago, IL] | 66004-1-Ig |
| **Flag®-tag** | **IB** | IB, 1:1000 | Sigma Aldrich [St. Louis, MO] | F1804 |
| **Anti-FLAG^®^ M2 Magnetic Beads** | **ChIP** | ChIP, 1:15 | Sigma Aldrich [St. Louis, MO] | M8823 |
| **HRP-β-actin** | **IB** | IB, 1:200000 | PTG Labs [Chicago, IL] | HRP-60008 |
| **HRP-GAPDH** | **IB** | IB, 1:200000 | PTG Labs [Chicago, IL] | HRP-60004 |
| **Normal Rabbit IgG** | **ChIP** | ChIP, 1:100 | EMD Millipore [Billerica, MA] | 12-370 |
| **Anti-Rabbit IgG HRP** | **IB** | IB, 1:5000 | GE Healthcare Bio-Sciences [Piscataway, NJ] | PI32460 |
| **Anti-Mouse IgG HRP** | **IB** | IB, 1:5000 | GE Healthcare Bio-Sciences [Piscataway, NJ] | 32430 |

**Table S2.** QPCR Primer sequences used, Related to the Materials and methods.

| **Gene Name** | **Forward primer** | **Reverse primer** |
| --- | --- | --- |
| **SUB1** | TTCGAGAGCCCTGTCATCTT | TTGCCTTTAAAATCGCGAAC |
| **PLK1** | GCCCCTCACAGTCCTCAATA | AGTCGACCACCTCACCTGTC |
| **BUB1B** | GTGCTTCCCAGTTTCACTCC | CCAGGCTTTCTGGTGCTTAG |
| **CDKN1B**  **DDIT4**  **STC1** | AAGAAGCCTGGCCTCAGAAG  CCTGGACAGCAGCAACAGT  CCTGAAGCCATCACTGAGGT | TTCATCAAGCAGTGATGTATCTGA  GGTCACTGAGCAGCTCGAA  ATCACATTCCAGCAGGCTTC |
| **GAPDH** | TGCACCACCAACTGCTTAGC | GGCATGGACTGTGGTCATGAG |
| **b-ACTIN**  **Human Alu**  **Human Alu** | GCACAGAGCCTCGCCTT  GTCAGGAGATCGAGACCATCCT  **Taqman probe** | GTTGTCGACGACGAGCG  AGTGGCGCAATCTCGGC  5′-6-FAM-AGCTACTCGGGAGGCTGAGGCAGGA-TAMRA-3′ |
|  |  |  |
|  |  |  |
|  |  |  |
|  |  |  |

**Table S3.** QPCR Primer sequences used either for SUB1 or Flag-SUB1 chromatin immunoprecipitation, Related to the Materials and methods.

| **Gene Name** | **Primer Name** | **Forward primer** | **Reverse primer** |
| --- | --- | --- | --- |
| **PLK1** | Site 1  Site 2 | GGAGAAACCCCGAAGGAAT  GCCTTTGCGGTTCTAACAAG | GGGAAAACCTGATTGACACG  AAGCTCCTGCGGTTCACTT |
| **C-MYC** | Site 1  Site 2 | CCAACAAATGCAATGGGAGT  CAGGAGGGGCGGTATCTG | GGAGGAAAACGATGCCTAGA  TGTATTATGCATTATGTATGCACAGC |
| **BUB1B** | Site 1  Site 2 | GCCATTGAATCCCAAAAACT  CCCAACACTCAAAACAGCAA | CTCCGTGCTCTCGCGTCT  AGCAGGCTTAGGCAAAACAA |

**Supplementary Figure legends:**

**Supplementary Figure S1.** QPCR analysis of *SUB1*, *DDIT4* and *STC1* in miR-101 treated cells. QPCR was performed in DU145 and PC3 cells treated either with miR-101 or non-targeting miRNA control. *ACTB* is used as an internal control. All bar graphs are shown with ± SEM.

**Supplementary Figure S2.** miR-101 binding sites in SUB1 3’-UTR. Schematic representation of (**a**) mutant-1 (M1) and **(b)** mutant-2 (M2) SUB1 3’-UTRs. Red nucleotides represent mutations that are predicted to disrupt potential miR-101 binding.

**Supplementary Figure S3.** QPCR analysis of *SUB1* in knockdown cells. (**a-g**) qPCR analyses were performed in prostate cancer cells treated either with SUB1 si/shRNA duplex or non-targeting si/shRNA. *ACTB* was used as an internal control. All bar graphs are shown with ± SEM.

**Supplementary Figure S4.** SUB1 knockdown inhibits wound-induced migration in prostate cancer cells**.** **(a)** Immunoblot analysis of SUB1 in stable DU145 and PC3-SUB1 knockdowns. β-actin was used as a loading control. **(b, c)** Wound healing assay in stable DU145 and PC3-SUB1 knockdown cells. SUB1 knockdown inhibited cell migration in wound healing assays of DU145 and PC3 cell lines at 24 h. Images were taken at 0 and 24 h after wound. The white lines show the margin of scratched area in which double headed arrow indicates scratch width (S) and white arrow indicates complete healing (R) of scratch wound.

**Supplementary Figure S5.** QPCR analysis of *SUB1* targets in modulated prostate cells. QPCR for (**a, b**) *PLK1*, (**c, d**) *BUB1B* and (**e-h**) *CDKN1B* was performed in prostate cells. *ACTB* was used as an internal control. All bar graphs are shown with ± SEM.

**Supplementary Figure S6.** Immunoblot analysis of CDKN1B in (**a**) DU145, (**b**) PC3-SUB1 knockdowns and (**c**) lenti stable RWPE-SUB1 cells. β-actin was used as a loading control.

**Supplementary Figure S7.** PLK1 and BUB1B are overexpressed in prostate cancer. **(a, b)** Expression levels of *PLK1* and *BUB1B* in normal prostate, primary tumor and metastatic tumor samples from TCGA. (**c**) Quantitative real-time PCR of *PLK1* using RNA from benign, prostate carcinoma (PCa) and metastatic prostate cancer (MET) tissues. *ACTB* was used as an internal control. (**d**) Immunoblot analysis of PLK1 protein expression in prostate tissue extracts using PLK1 antibody. β-actin was used as a loading control. QPCR analysis of (**e**) *SUB1* and (**f**) *PLK1* in prostate cancer cell lines. All bar graphs are shown with ± SEM.

**Supplementary Figure S8.** SUB1 stable over-expression markedly increases cell proliferation in RWPE cells. Immunoblot analysis showing SUB1 and DDK-tag (Myc-DDK-SUB1) in RWPE cells (Inset). Stable RWPE-SUB1 over-expressing cells showed increased cell proliferation than untreated or lacZ over-expressing cells.
