# Supplementary figures and images for: MicroRNA-101 regulated transcriptional modulator SUB1 plays a role in prostate cancer
Source: Oncogene. 2016 Jun 6;35(49):6330–40. doi: 10.1038/onc.2016.164 (PMC5140777; doi:10.1038/onc.2016.164)

# Supplementary Figure S1

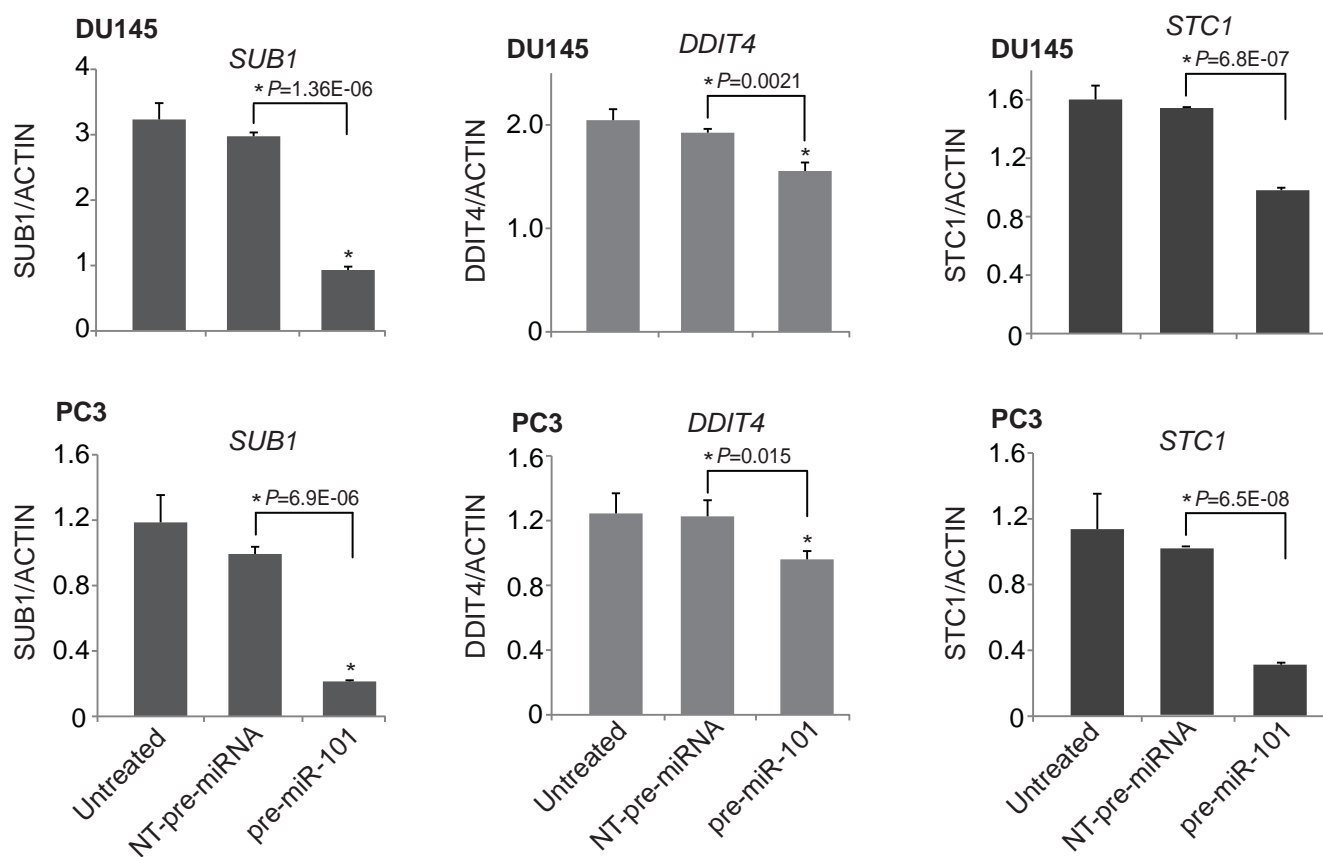

Supplement: Supplementary Figure 1 [file onc2016164x2.pdf]

# Supplementary Figure S2

**a**

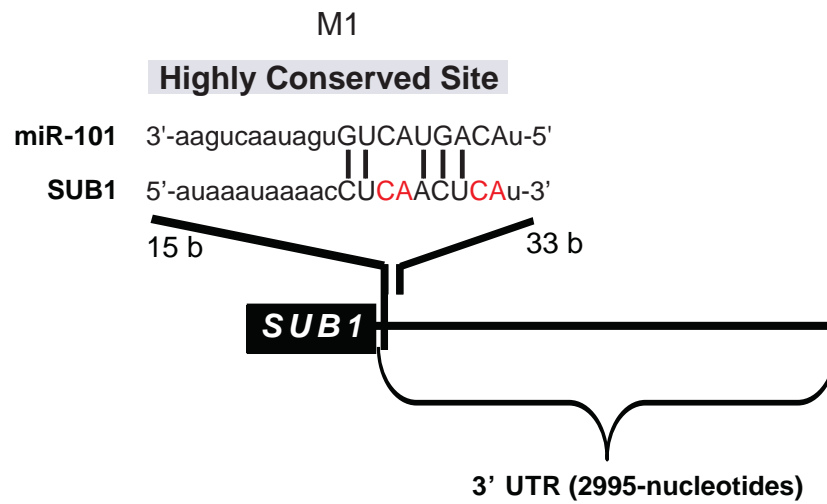

**b**

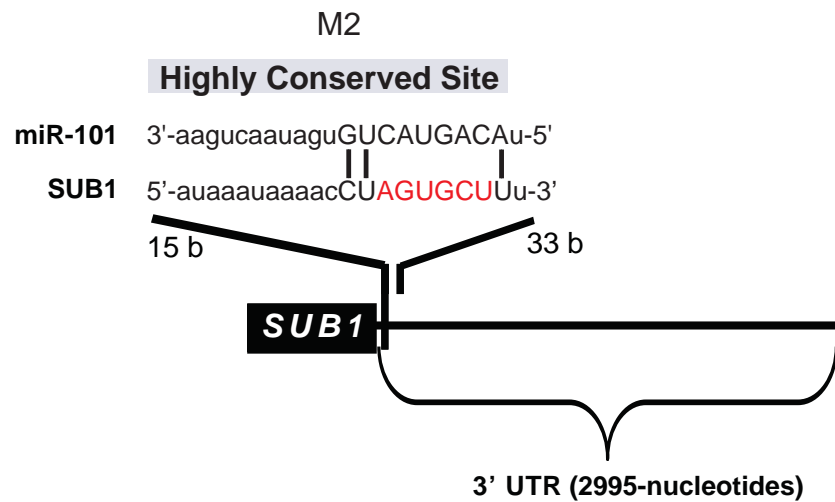

Supplement: Supplementary Figure 2 [file onc2016164x3.pdf]

# Supplementary Figure S3

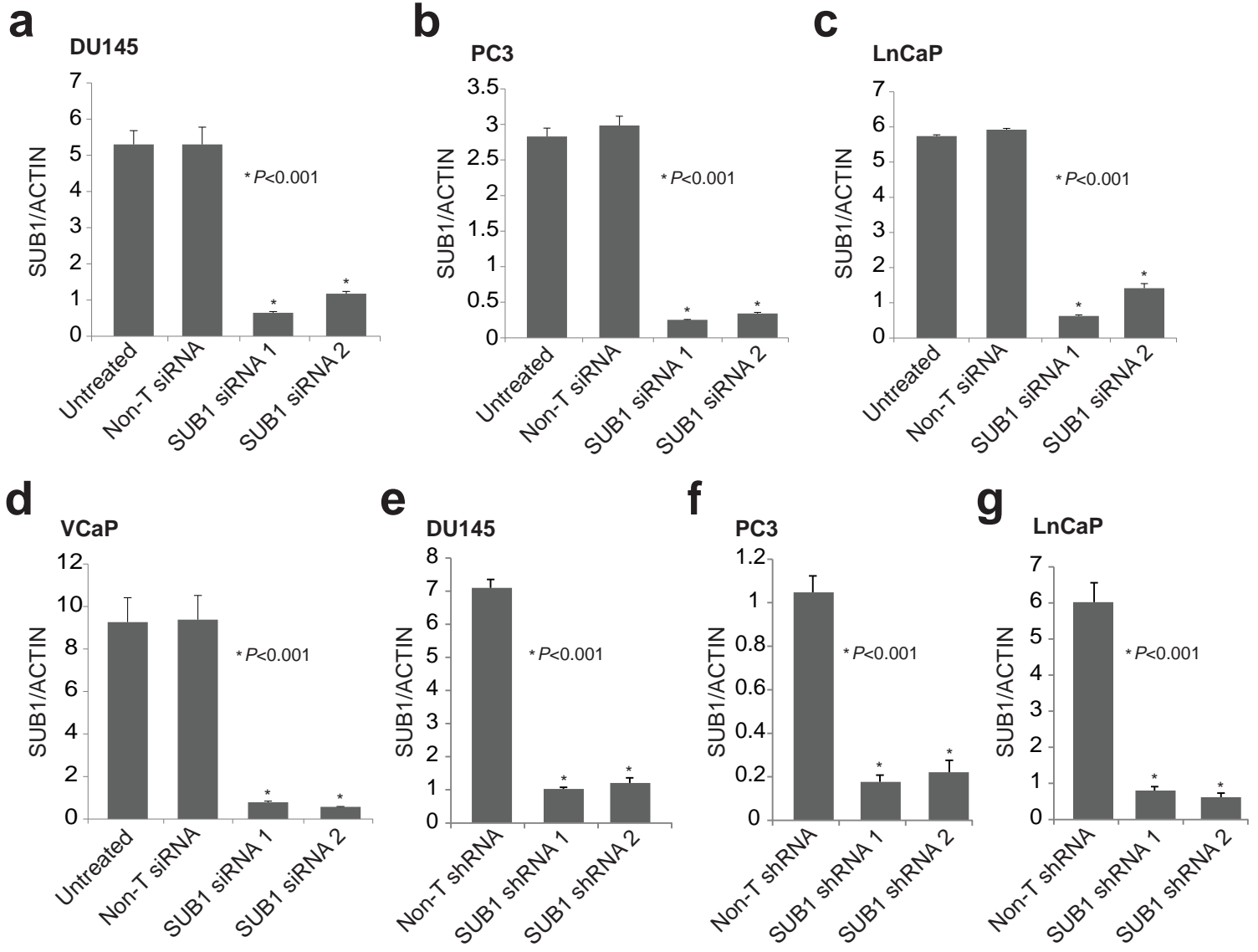

Supplement: Supplementary Figure 3 [file onc2016164x4.pdf]

# Supplementary Figure S4

**a**

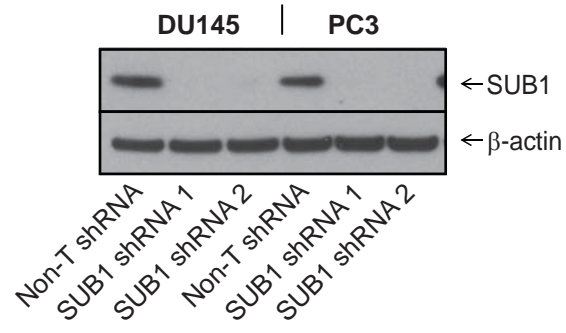

**b**

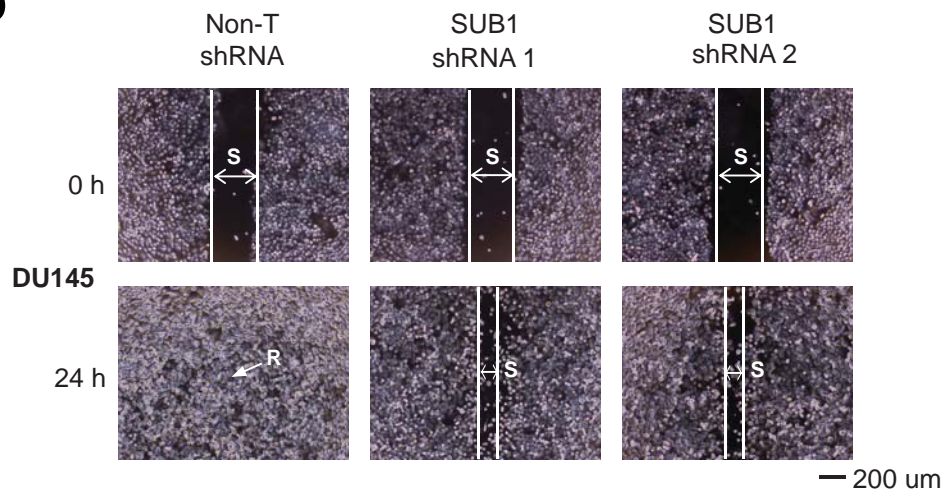

**c**

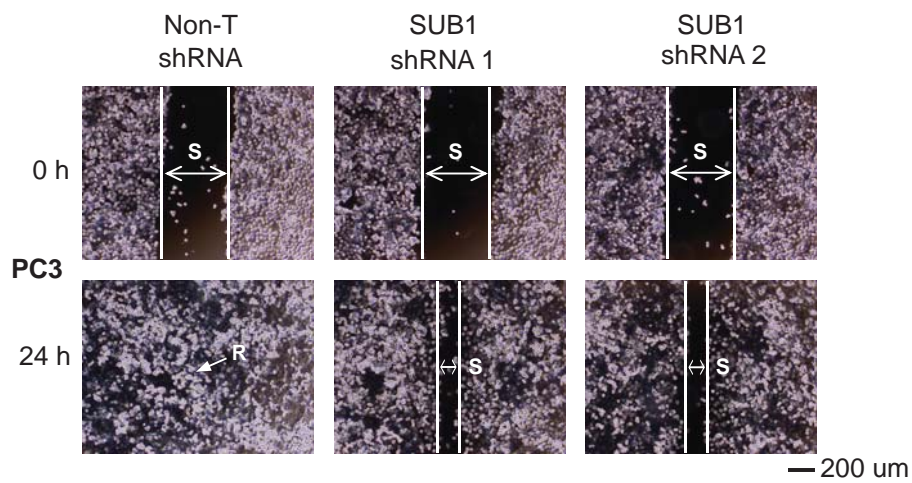

Supplement: Supplementary Figure 4 [file onc2016164x5.pdf]

# Supplementary Figure S5

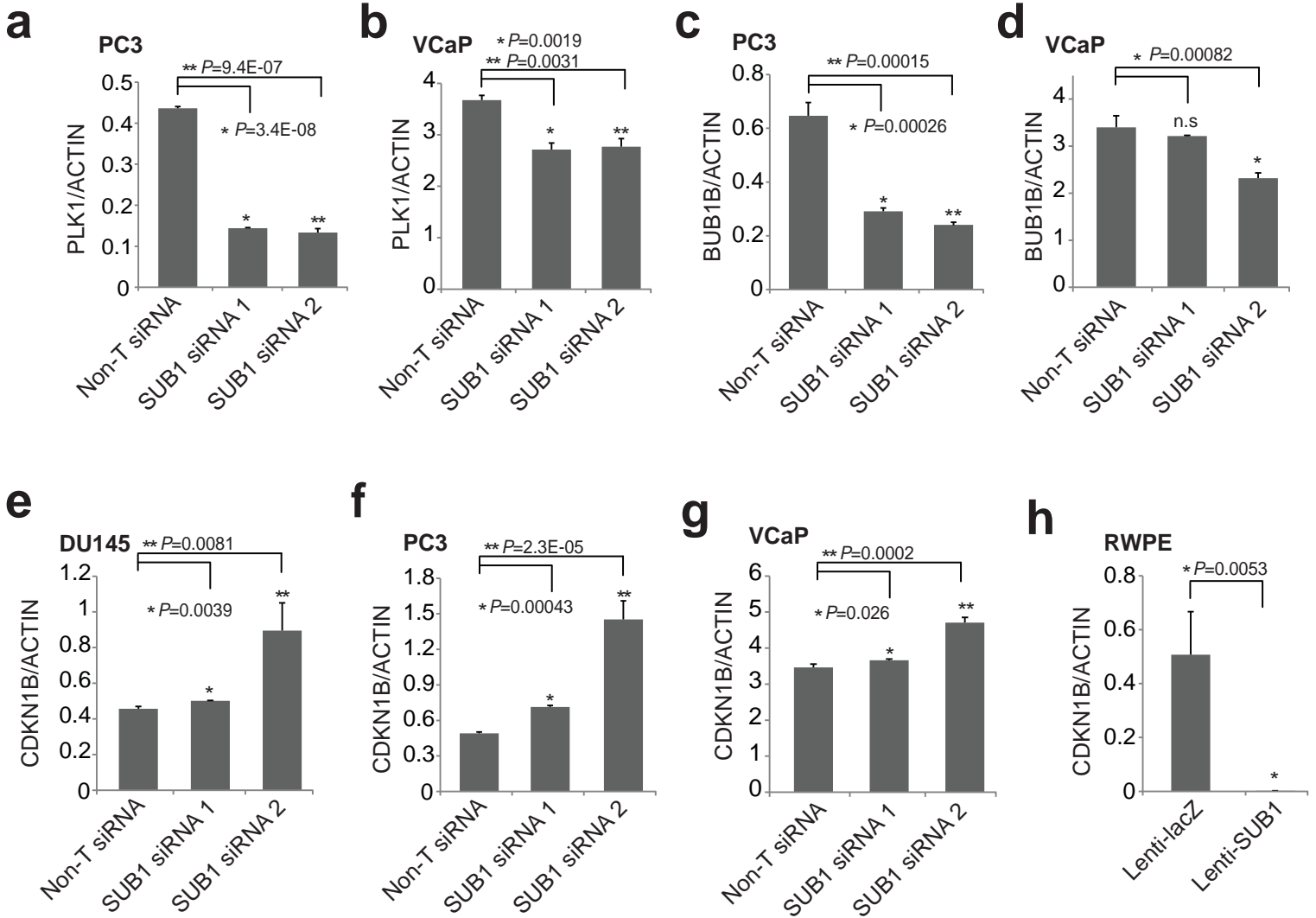

Supplement: Supplementary Figure 5 [file onc2016164x6.pdf]

## Supplementary Figure S6

**a**

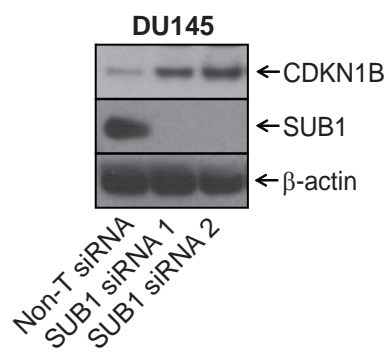

**b**

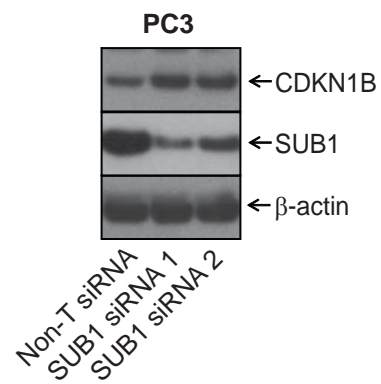

**c**

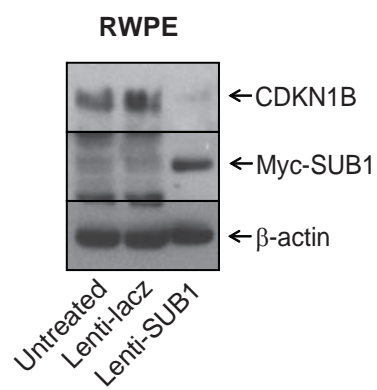

Supplement: Supplementary Figure 6 [file onc2016164x7.pdf]

# Supplementary Figure S7

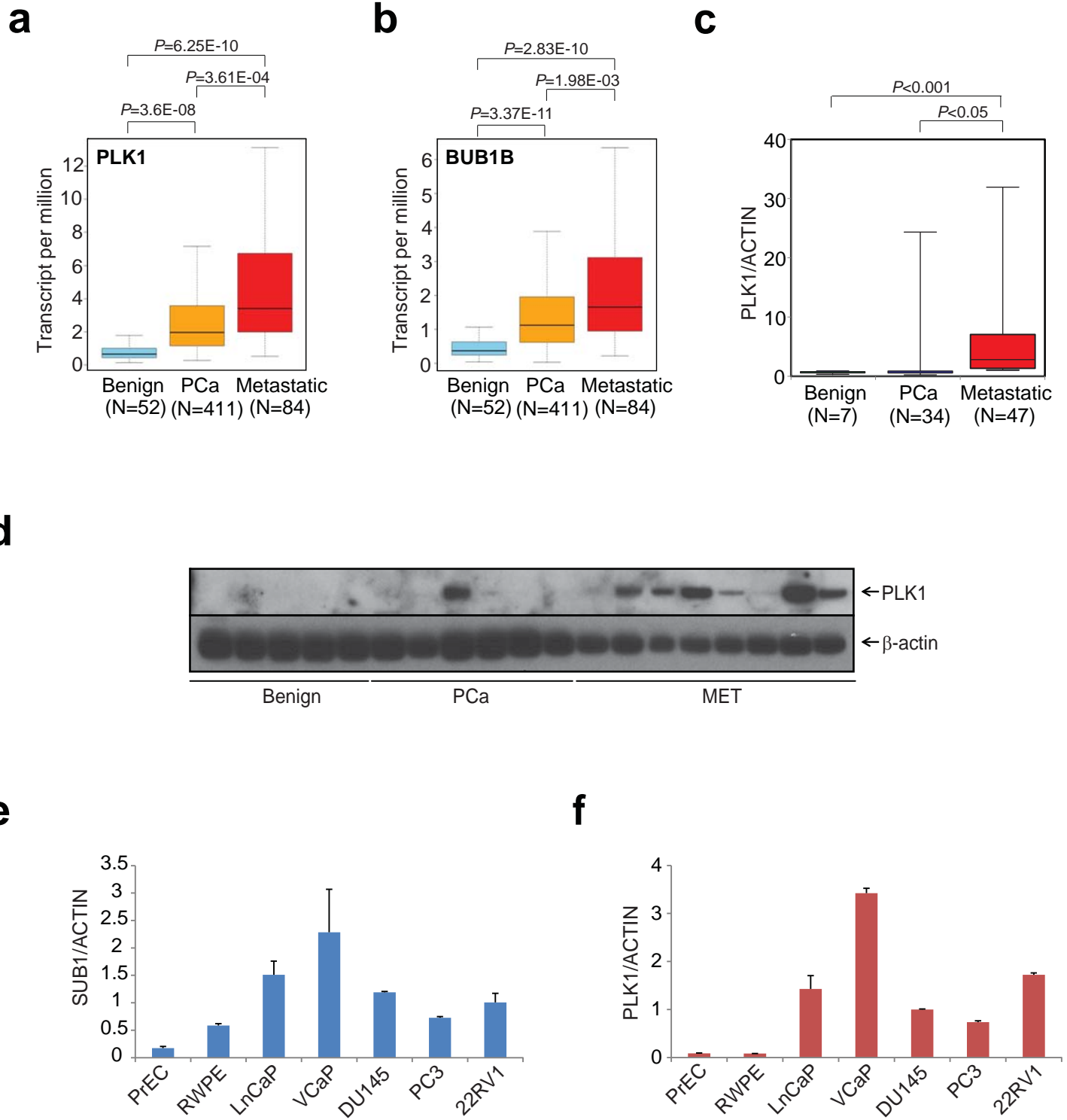

Supplement: Supplementary Figure 7 [file onc2016164x8.pdf]

## Supplementary Figure S8

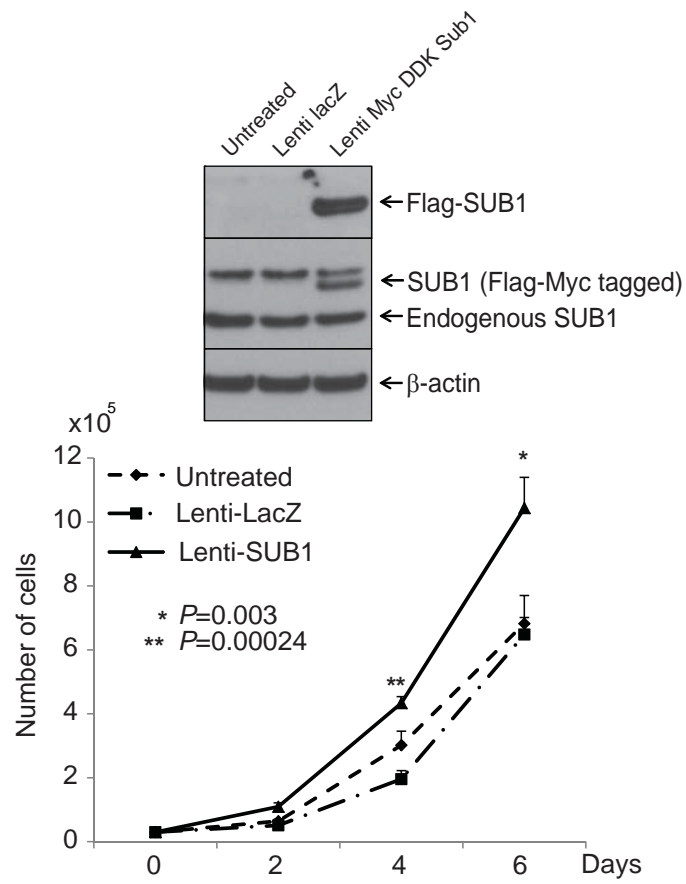

Supplement: Supplementary Figure 8 [file onc2016164x9.pdf]
